# Virus-induced gene silencing as a tool for functional analyses in the emerging model plant *Aquilegia *(columbine, Ranunculaceae)

**DOI:** 10.1186/1746-4811-3-6

**Published:** 2007-04-12

**Authors:** Billie Gould, Elena M Kramer

**Affiliations:** 1 Department of Organismic and Evolutionary Biology, Harvard University, 16 Divinity Ave, Cambridge, MA, 02138 USA

## Abstract

**Background:**

The lower eudicot genus *Aquilegia*, commonly known as columbine, is currently the subject of extensive genetic and genomic research aimed at developing this taxon as a new model for the study of ecology and evolution. The ability to perform functional genetic analyses is a critical component of this development process and ultimately has the potential to provide insight into the genetic basis for the evolution of a wide array of traits that differentiate flowering plants. *Aquilegia *is of particular interest due to both its recent evolutionary history, which involves a rapid adaptive radiation, and its intermediate phylogenetic position between core eudicot (e.g., *Arabidopsis*) and grass (e.g., *Oryza*) model species.

**Results:**

Here we demonstrate the effective use of a reverse genetic technique, virus-induced gene silencing (VIGS), to study gene function in this emerging model plant. Using *Agrobacterium *mediated transfer of tobacco rattle virus (TRV) based vectors, we induce silencing of *PHYTOENE DESATURASE *(*AqPDS*) in *Aquilegia vulgaris *seedlings, and *ANTHOCYANIDIN SYNTHASE *(*AqANS*) and the B-class floral organ identity gene *PISTILLATA *in *A. vulgaris *flowers. For all of these genes, silencing phenotypes are associated with consistent reduction in endogenous transcript levels. In addition, we show that silencing of *AqANS *has no effect on overall floral morphology and is therefore a suitable marker for the identification of silenced flowers in dual-locus silencing experiments.

**Conclusion:**

Our results show that TRV-VIGS in *Aquilegia vulgaris *allows data to be rapidly obtained and can be reproduced with effective survival and silencing rates. Furthermore, this method can successfully be used to evaluate the function of early-acting developmental genes. In the future, data derived from VIGS analyses will be combined with large-scale sequencing and microarray experiments already underway in order to address both recent and ancient evolutionary questions.

## Background

The genus *Aquilegia *is comprised of approximately 70 species distributed across temperate areas of North America, Europe, and Asia, with several ornamental varieties sold commercially [1]. These species have undergone a very recent and rapid adaptive radiation in response to biotic and abiotic factors, resulting in low sequence variation among species [2-4]. Thus, they are ideal for evolutionary studies in that they display a wide range of ecological and morphological diversity but retain high levels of cross-compatibility between species, allowing for genetic dissection of traits [5]. *Aquilegia *possesses a small diploid genome (n = 7, 1C = 320–400 Mbp, S. Hodges, pers. comm.), is self-fertile and reproduces regularly with high fecundity. In addition, *Aquilegia *occupies an intermediate phylogenetic position between core eudicot (e.g., *Arabidopsis*) and grass (e.g., *Oryza*) model species. Thus functional genetic analyses of physiological and morphological adaptation in *Aquilegia *will be valuable in making evolutionary comparisons across divergent angiosperms. Many resources already exist to facilitate research in this genus, including genetic maps of several major quantitative trait loci [5], a fingerprinted BAC library [6], and an annotated expressed sequence tag (EST) database [7]. Most significantly, sequencing of the entire *Aquilegia *genome will commence at the Joint Genome Institute in 2007 [8]. To add to this growing body of research, here we demonstrate effective use of a rapid reverse genetic technique, virus-induced gene silencing (VIGS), in this emerging model plant.

VIGS is a method that utilizes the RNAi pathway in plants to induce transient gene knock-down [9]. This process begins with the *Agrobacterium*-mediated introduction of modified virus-based cDNA constructs that also contain fragments of endogenous gene sequences. Once expressed *in vivo*, dsRNAs are generated from an encoded viral polymerase as the virus replicates and spreads through the plant (reviewed [10]). These dsRNAs are then targeted by DICER-like enzymes and degraded into siRNA. In turn, the siRNA molecules provide a template for degradation of complimentary RNAs, including complimentary endogenous mRNAs, by the RNA-induced silencing complex (RISC). Silencing persists until proliferation of viral RNAs is overcome by the silencing response.

**Figure 1:**
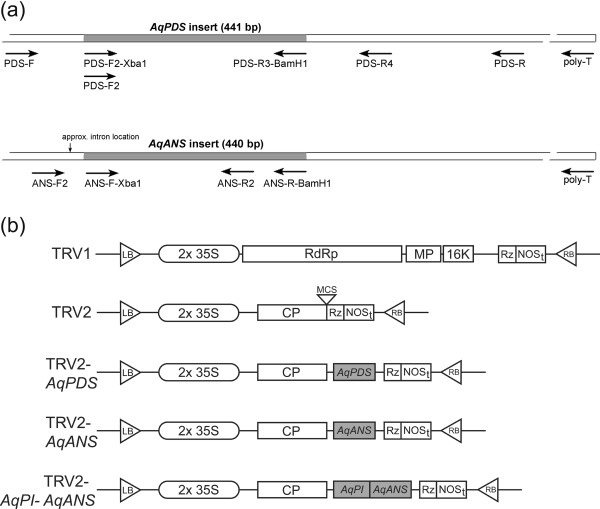
**VIGS Constructs**. (a) Schematic of the *AqPDS *and *AqANS *cDNAs with primer positions indicated. (b) Schematic of TRV1 and 2 constructs: LB, left border; RB, right border; RdRp, RNA-dependant RNA polymerase; MP, movement protein; 16 K, 16 Kd protein; Rz, self-cleaving ribozyme; NOSt, NOS terminator; CP, coat protein; MCS, multiple cloning site.

Induction of VIGS is a useful alternative to the often difficult and laborious process of generating stably transformed plants, and offers the ability to overcome functional redundancy by suppressing all or most members of a gene family [11]. VIGS vectors have been developed from viral systems that affect many plant hosts including many dicots (reviewed [10-12]) and several monocots (barley [13] and wheat [14]). Here we demonstrate VIGS in the species *Aquilegia vulgaris *using vectors based on the bipartite genome of the tobacco rattle virus (TRV) (reviewed [9]). This system uses two vectors, derived from binary transformation plasmids, which have cDNAs encoding the TRV RNA1 (TRV1) and TRV RNA2 (TRV2) inserted into the T-DNA region [15]. Both vectors contain a duplicated 35S promoter and a self-cleaving ribozyme sequence to enable rapid generation of intact viral transcripts (Figure 1). Genes essential for plant to plant transmission of TRV through its nematode vector [16] have been deleted from TRV2 [15]. The TRV system has the advantage of being able to penetrate meristematic cells [11] and overall causes mild viral symptoms in a wide range of susceptible hosts [17]. Although TRV-VIGS has primarily been used in the Solanaceae (tobacco [18]), it has also been successfully applied in *Arabidopsis *[19] and the lower eudicot *Papaver *(poppy [20]).

Here we have successfully produced TRV-VIGS of two phenotypic marker genes in *Aquilegia: PHYTOENE DESATURASE *(*AqPDS*) and *ANTHOCYANIDIN SYNTHASE *(*AqANS*). Silencing of *AqPDS *results in decreased production of photoprotective carotenoid proteins and the subsequent breakdown of chlorophyll pigments [21,22]. This is easily detectable as a photobleaching phenotype in chlorophyll-containing tissues. Silencing of *AqANS *reduces conversion of colorless leucoanthocyanidins to colored anthocyanidin [23] inhibiting development of the purple wild-type flower color. We have compared *AqPDS *silencing at two different developmental stages, using RT-PCR to detect viral transcripts in silenced plants and the simultaneous decrease in the relative expression level of endogenous *AqPDS *transcript. Similarly we have measured endogenous *AqANS *levels in silenced and unsilenced *Aquilegia *flowers. Finally, we have used TRV-VIGS to simultaneously knock-down *AqANS *and a non-marker locus, the floral developmental gene *PISTILLATA *[24,25], in order to generate a homeotic floral phenotype. This study demonstrates that VIGS will be a useful tool for analyzing a wide range of gene function in *Aquilegia *that can now be combined with other genetic tools for functional studies in this genus.

## Results and Discussion

### TRV-*AqPDS*-VIGS treatment of *Aquilegia vulgaris *seedlings

In order to test the ability of TRV-based VIGS to promote gene silencing in *Aquilegia*, we prepared a TRV2 construct [9] containing a 441 bp fragment of *AqPDS *(Figure 1a). This fragment was initially isolated from *A. vulgaris *cDNA using degenerate primers and then re-amplified using locus-specific primers for insertion into the TRV2 plasmid. As discussed above, VIGS-induced silencing of the endogenous *AqPDS *locus should result in an easily recognizable photobleached phenotype. Although we tested several methods for introducing the TRV1/2 plasmids, including direct injection of roots and vegetative parts (data not shown), only vacuum infiltration of seedlings yielded strong and consistent silencing of *AqPDS*. Thus far we have been successful in inducing TRV-VIGS in three species, *A. vulgaris*,*A. caerulea*, and *A. alpina*, but for this study we focused on *A. vulgaris*. TRV-VIGS treatment was carried out on seedlings in two developmental stages: those with 1–2 true leaves and those with 3–5 true leaves. Cotyledon-stage seedlings were not used because initial trials showed high rates of mortality. Groups of seedlings were treated with the TRV1 and TRV2-*AqPDS *constructs or with TRV1 and TRV2 (unmodified) as controls (Figure 1b). Four independent trials were performed for each treatment.

**Figure 2:**
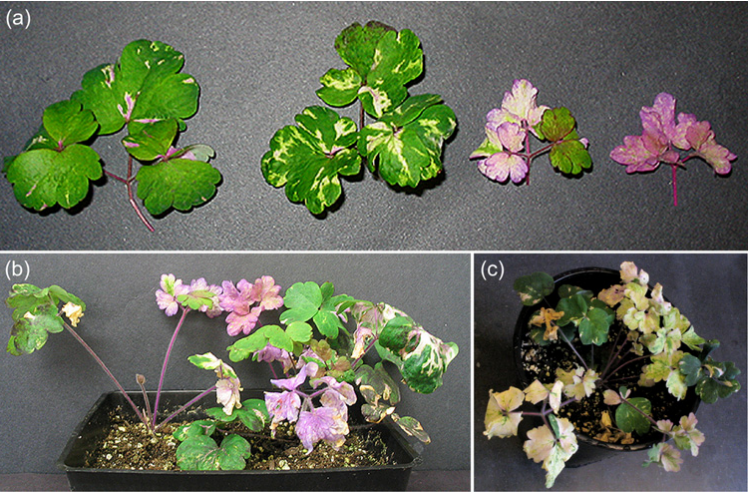
***AqPDS *silencing phenotypes**. (a) Variation in *AqPDS *silencing phenotypes in the leaves of *TRV2-AqPDS *treated *Aquilegia *seedlings. (b) Whole seedlings exhibiting photobleaching 3–4 weeks post-treatment with TRV1 and TRV2-*AqPDS*. (c) Plant exhibiting strong photobleached phenotype 3–4 months post-treatment with TRV2-*AqPDS*. Weakly photobleached leaves represent the first leaves that arose after treatment. These leaves were maintained by the plant while later arising leaves, which showed strong photobleaching, senesced and were subsequently removed. The most recent leaves, still exhibiting strong photobleaching, are present.

Among 406 *A. vulgaris *seedlings treated with the *AqPDS *silencing construct, survival rates were higher when plants were treated at the 3–5 true leaf stage (64% survival) versus the 1–2 leaf stage (49% survival). Survival rates among 229 mock treated control plants were comparable to experimentally treated ones in both groups (Table 1). In terms of silencing response, a slightly higher frequency of the VIGS phenotype was observed in the 1–2 leaf seedling group (11.9% of treated plants) versus the 3–5 leaf group (9.6%). No photobleaching was observed in the mock treated control group (Table 1). Of the seedlings that exhibited the photobleached VIGS phenotype, the extent of silencing ranged from mild to strong, from leaves displaying a partial mosaic to those that were completely white (Figure 2a). Many of the plants showing the strongest response exhibited persistent photobleaching of newly arising leaves for up to four months (Figure 2b, c). These plants did not maintain silencing through vernalization treatment, however (data not shown).

**Table 1:** Survival and silencing rates among seedlings

Seedling size, Construct	N Treated	% Survival	% Phenotype (of total)	% Phenotype (of survivors)
1–2 L^a^, TRV2-*AqPDS*	218	48.6%	11.9%	24.5%
1–2 L, TRV2	97	52.6%	0.0%	0.0%
3–5 L^b^, TRV2-*AqPDS*	188	64.4%	9.6%	14.9%
3–5 L, TRV2	132	59.1%	0.0%	0.0%

^a^1–2 L = 1–2 leaves at infiltration; ^b^3–5 L = 3–5 leaves at infiltration.

**Figure 3:**
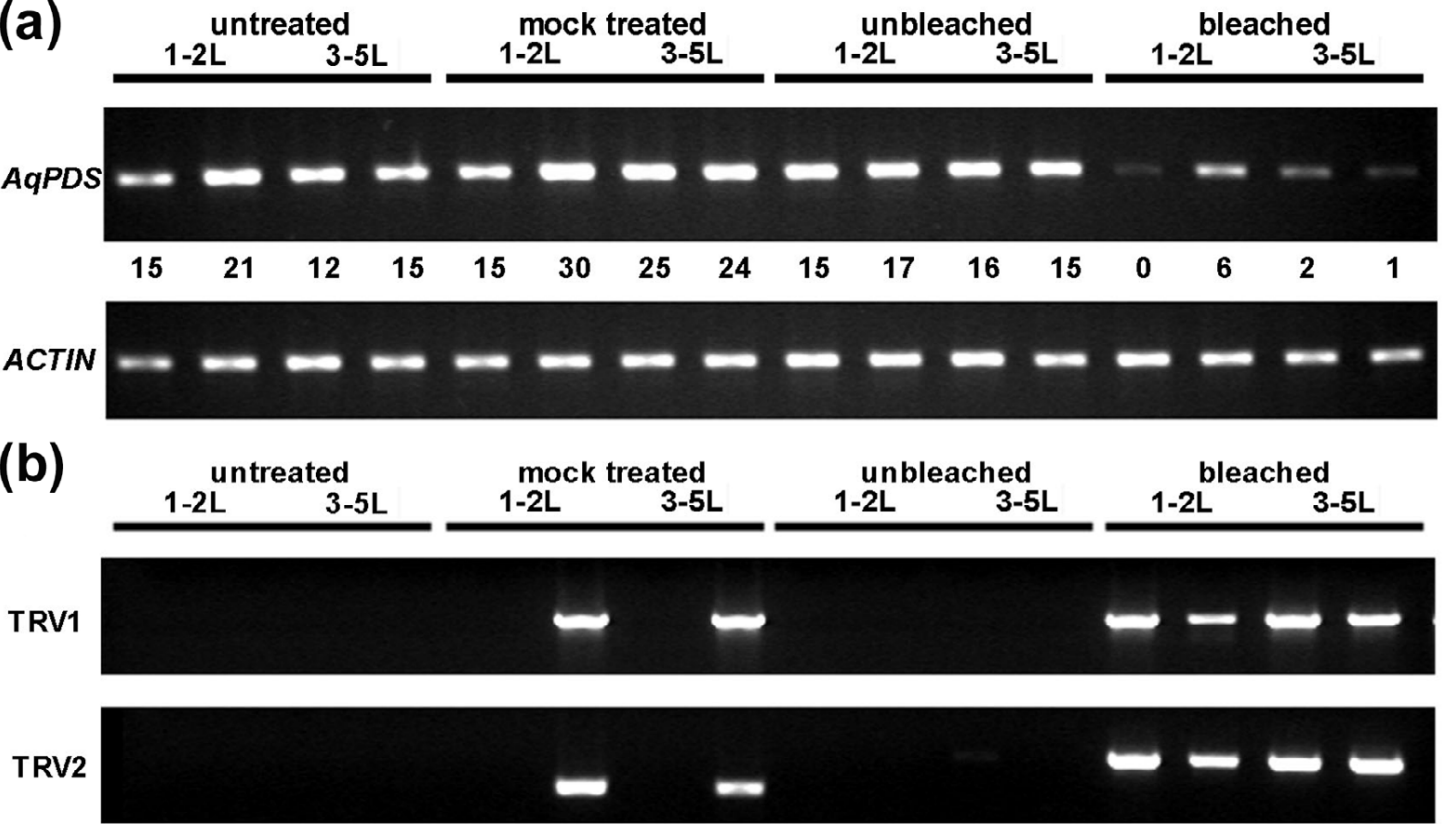
**Endogenous expression levels of *AqPDS *and TRV1/TRV2**. (a) Semi-quantitative RT-PCR of *AqPDS *expression for selected untreated, mock treated, treated unbleached, and treated photobleached seedlings. Seedlings had 1–2 true leaves (1–2L) or 3–5 true leaves (3–5L) at the time of treatment. Normalized values for the *AqPDS*/*ACTIN *ratios are shown for each sample. A value of 0.0 may indicate that the band was below the detection limit of the imaging system. (b) RT-PCR detection of TRV1 and TRV2 viral RNAs in selected untreated, mock treated, treated unbleached, and treated photobleached seedlings.

In order to confirm that observed photobleaching was due to infection with TRV1 and TRV2 as well as silencing of the endogenous *AqPDS *locus, we performed semi-quantitative RT-PCR on all treatment groups, comparing bleached and unbleached individuals (Figure 3 and Table 2). The presence of both TRV RNAs was detected in 100% of sampled plants exhibiting a bleached phenotype and in 0% of untreated plants. Among sampled plants that were treated with the silencing construct but that did not exhibit a bleached phenotype, 96% showed no presence of either TRV1 or TRV2. One plant in this group was positive for both RNAs indicating that infection had occurred but did not produce silencing. In the mock treated control group, 69% of plants showed no presence of either TRV1 or TRV2, and 31% showed the presence of both, which is indicative of the approximate overall rate of uptake of these constructs in *A. vulgaris *seedlings. We did not detect only one of the viral RNAs in any of the samples.

**Table 2:** RT-PCR Detection of TRV1 and TRV2 RNAs in TRV2-*AqPDS *experimental plants

	Untreated, n = 11	Infiltrated with TRV1 and TRV2-*AqPDS*; photobleached, n = 17	Infiltrated with TRV1 and TRV2-*AqPDS*; unbleached, n = 17	Infiltrated with TRV1 and TRV2; unbleached, n = 16
TRV1 & TRV2*	0%	100%	6%	31%
No TRV	100%	0%	94%	69%

*The presence of only one of the TRV RNAs was not detected in any sample.

**Figure 4:**
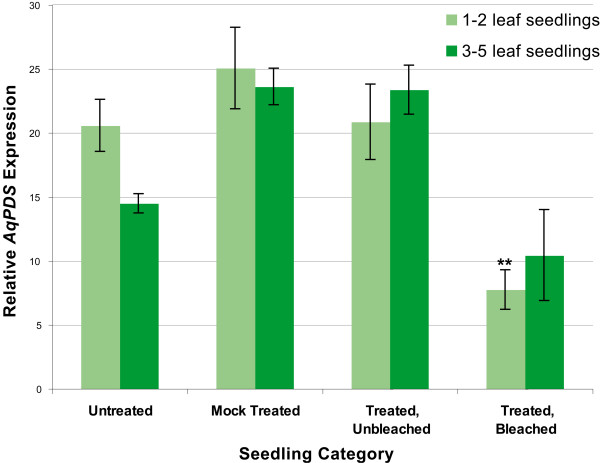
**Relative expression levels of endogenous *AqPDS *transcripts in TRV-VIGS-*AqPDS *treatment groups**. 7–9 plants were sampled in each category for plants with 1–2 leaves or 3–5 leaves. Sampling of leaves in the bleached category included tissue from a range of phenotypes (Figure 2). Asterisks indicate statistically significant difference when compared to Untreated expression levels. Standard error bars are shown.

Semi-quantitative RT-PCR supported the conclusion that the observed photobleached phenotype was caused by a reduction in the levels of endogenous *AqPDS *transcripts. Lower relative levels of expression were detected in photobleached plants compared with both untreated plants and mock treated plants from both seedling stages. In the 3–5 leaf seedling group, we detected an average 2.0 fold reduction in *AqPDS *levels between TRV-VIGS treated bleached seedlings and untreated seedlings. This difference was not statistically significant due to the high levels of variation in the degree of photobleaching. Reduction of *AqPDS *transcript levels in the 3–5 leaf group ranged from no detectable reduction up to a 15-fold decrease in expression as compared with the average *AqPDS *level in untreated seedlings. In the 1–2 leaf developmental stage group there was an average 2.7 fold reduction in the *AqPDS *level (Figure 4), which was much more consistent and highly significant at p < 0.0005.

### TRV-*AqANS*-VIGS treatment of adult *Aquilegia vulgaris *plants

**Figure 5:**
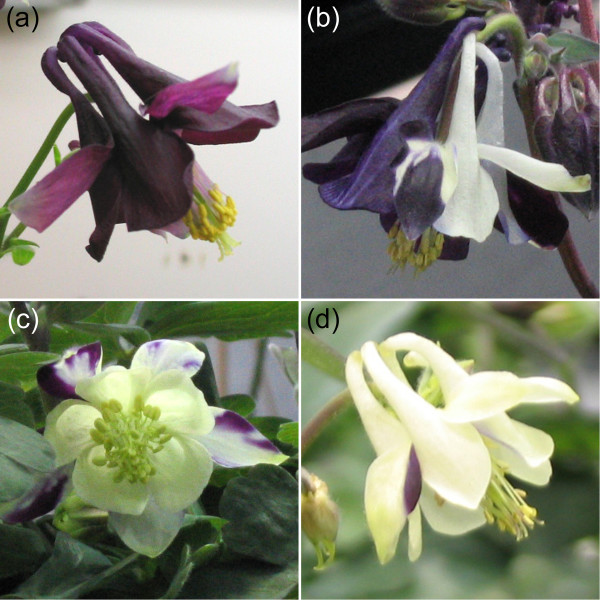
**Wildtype and *AqANS*-silenced flowers**. (a) Wild-type *Aquilegia vulgaris *flower. (b-d) Examples of mild to strong *AqANS *silencing in flowers of plants that were injected with TRV1 and TRV2-*AqANS *following vernalization.

In order to test if VIGS can be used to create silencing phenotypes in *Aquilegia *flowers, we targeted the *ANTHOCYANIDIN SYNTHASE (AqANS) *gene in post-vernalization (cold treated) plants. *AqANS *appears to be a single copy locus in *Aquilegia *(S. Hodges, pers. comm., [26]). The *AqANS *sequence was obtained from the recently compiled *Aquilegia *EST database and used to amplify and clone a 440 bp fragment into the TRV2 vector (see Experimental Procedures). Adult plants with 15 or more leaves were vernalized for 8 weeks at 4°C to induce flowering. Two weeks after transfer to the greenhouse at 20°C, we wounded each plant at the basal rosette and injected the mix of *Agrobacterium *solutions into the wound with a needle-less syringe at weekly intervals. We also tested floral dip-based methods [27] and vacuum infiltration of inflorescences, but, unlike the experiments with the seedlings, we had more consistent success with the injection method (data not shown). Two separate groups of plants were treated with the injection technique for a total of 48 plants treated with TRV1/TRV2-*AqANS*, 6 control plants treated with TRV1/TRV2 (unmodified), and 4 plants left untreated. Survival rates were 100% for both TRV treatment groups (one untreated plant expired of natural causes during the experiment) and there were only occasional instances of early floral senescence as a result of the treatment. About 3–4 weeks after treatment, depending on the speed of the onset of flowering, we observed a range of floral silencing phenotypes (Figure 5) in 12.5% of plants. In these plants, the number of *AqANS*-silenced flowers per plant varied from as few as two to as many as ten.

**Figure 6:**
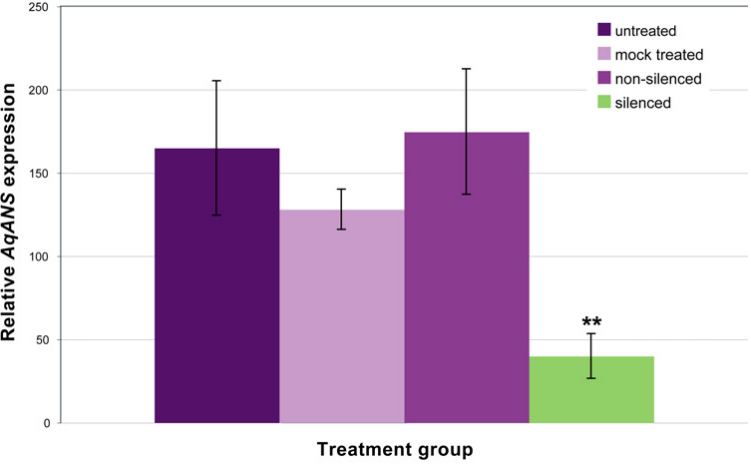
**Endogenous expression levels of *AqANS *and TRV1/TRV2**. (a) Semi-quantitative RT-PCR of *AqANS *expression for selected untreated, mock treated, treated unbleached, and treated bleached flowers. Normalized values for the *AqANS*/*ACTIN *ratios are shown for each sample. (b) RT-PCR detection of TRV1 and TRV2 transcripts for samples in each group.

**Figure 7:**
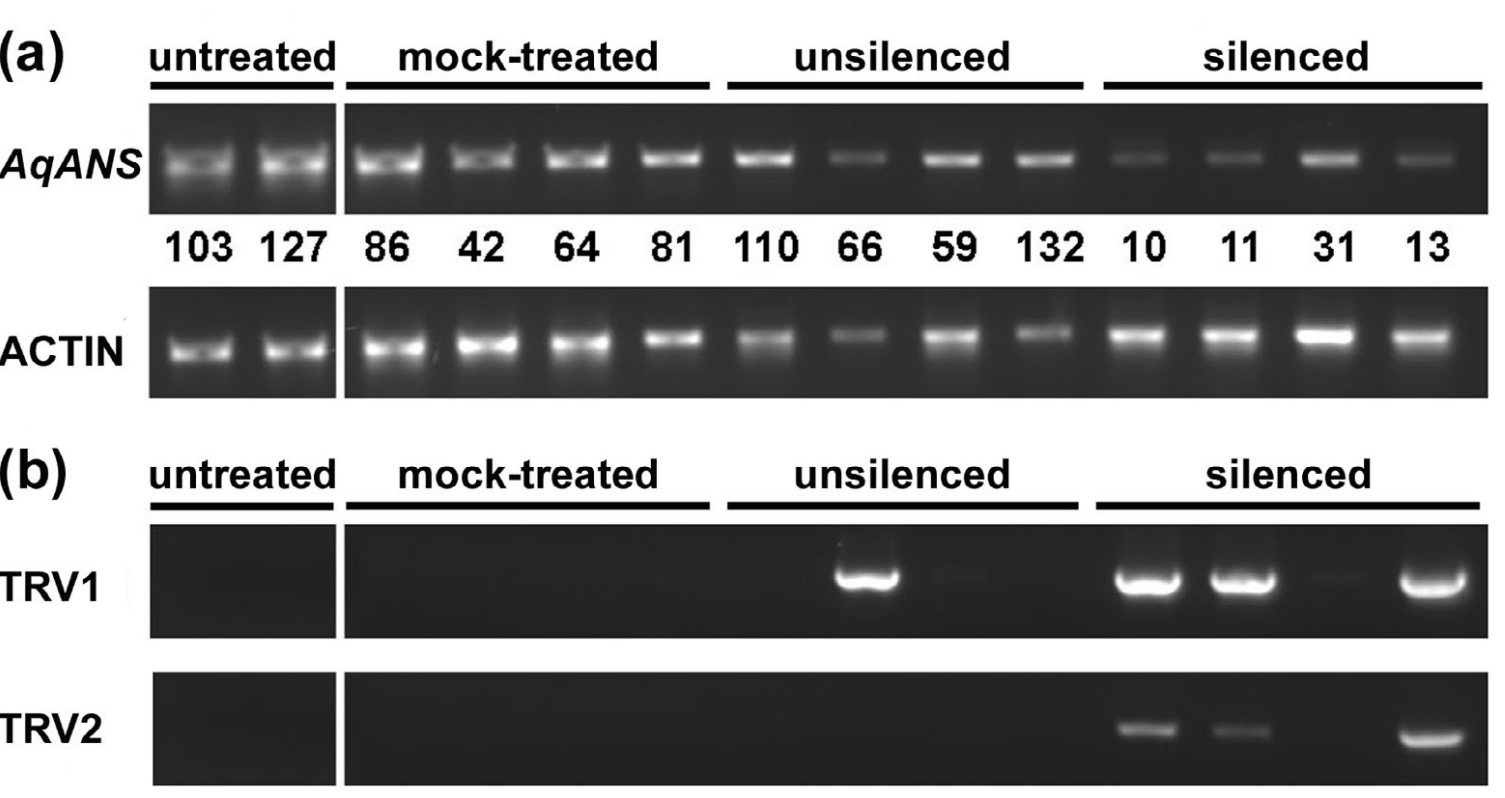
**Relative expression levels of endogenous *AqANS *transcripts in TRV-VIGS-*AqANS *treatment groups**. 8–10 samples were measured per category. Sampling of flowers in the silenced category included tissue from a range of phenotypes (Figure 5). Asterisks indicate statistically significant difference when compared to Untreated expression levels. Standard error bars are shown.

As with the *AqPDS *analysis, we collected tissue from a range of treatments and silencing phenotypes in the TRV2-*AqANS *silencing group. We conducted RT-PCR analysis to determine the levels of endogenous *AqANS *transcripts in the tissue and tested for the presence of TRV1 and TRV2. We found that *AqANS *transcript levels in white flowers were significantly reduced (p < 0.0005) an average of 4.1 fold, with some flowers showing up to an 8.3 fold reduction. *AqANS *levels in mock treated and treated but unsilenced flowers were both comparable to levels in untreated flowers (Figures 6 and 7).

We did not detect TRV1 or TRV2 transcripts in any untreated plants. We also did not detect any of these RNAs in a sample of 6 mock treated plants. This is likely due to the relatively low infection rate of the TRV2 and TRV1 constructs in adult plants. However, we did detect TRV1 and TRV2-*AqANS *transcripts in 78% of bleached tissue samples and we detected TRV1 in one treated unsilenced sample (Table 3, Figure 6). The lack of detection of viral transcripts in some of the silenced tissue samples likely indicates the persistence of *AqANS*-silencing even after TRV expression has been repressed. *AqANS*-silenced tissue was collected primarily from fully mature flowers, typically two or more weeks following their first injection, and hence some samples may have been collected after TRV had been suppressed to undetectable levels by the silencing response. Overall, the data supports the conclusion that the white floral phenotype was caused by introduction of the TRV1/TRV2-*AqANS *constructs during floral development.

**Table 3:** RT-PCR Detection of TRV1 and TRV2 RNAs in TRV2-*AqANS *experimental plants

	Untreated	Injected with TRV1 and TRV2-*AqANS*: Silenced	Injected with TRV1 and TRV2-*AqANS*: Unsilenced	Injected with TRV1 and TRV2 (Mock)
#plants sampled	4	5	7	6
#flowers sampled	4	9	8	8

TRV1 + TRV2	0%	78%	0%	0%
Neither	100%	22%	88%	100%
TRV1 only	0%	0%	12%	0%

**Figure 8:**
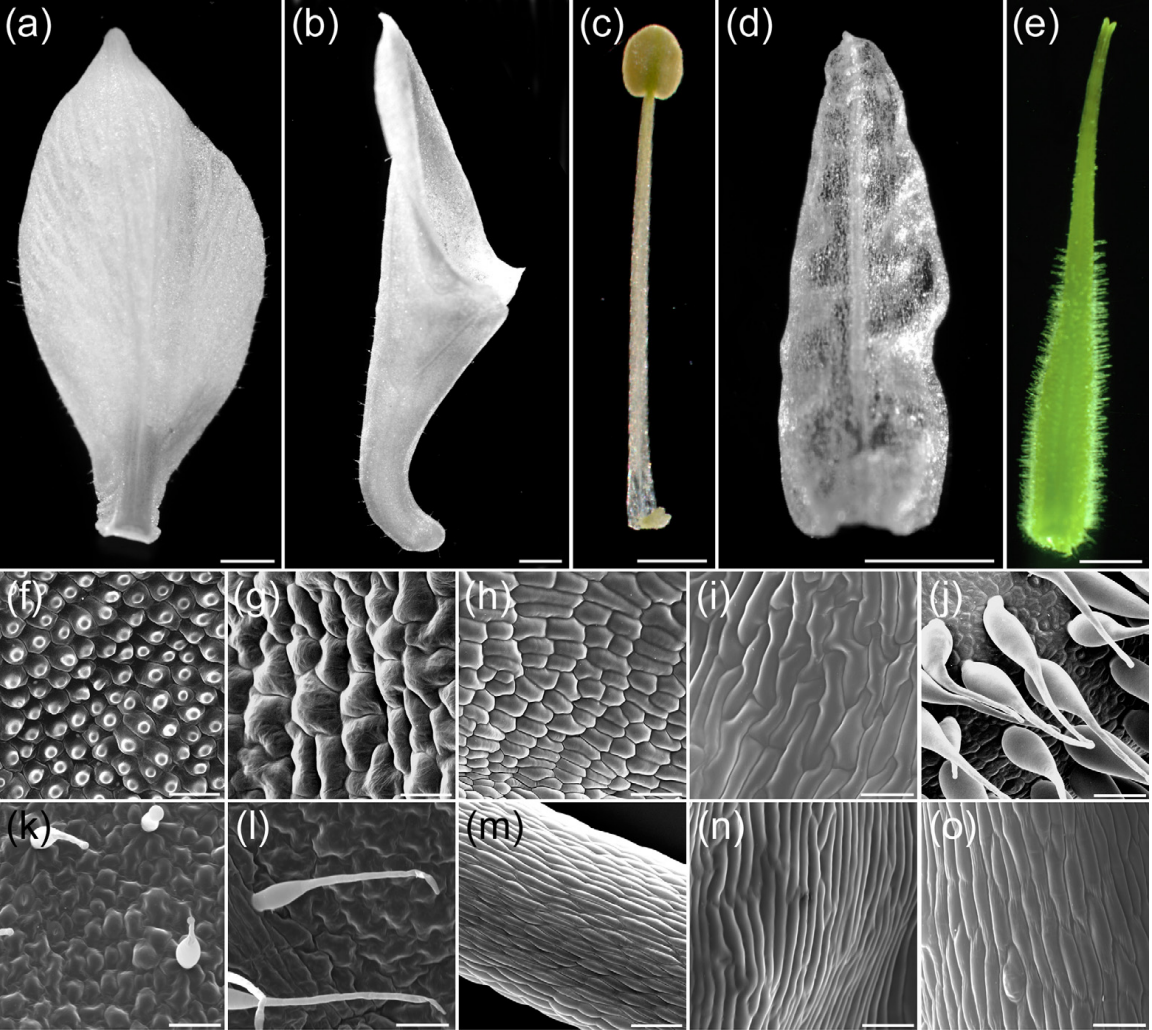
**Light micrographs of floral organs from *AqANS*-silenced flowers and SEM images of each organ type**. Light micrographs (a-e). SEM images (f-o). (a) Sepal. (b) Petal. (c) Stamen. (d) Staminodium. (e) Carpel. (f, k) Adaxial (f) and abaxial (k) surfaces of the sepal. (g, l) Adaxial (g) and abaxial (l) surfaces of the petal. (h, m) Anther (h) and filament (m) surfaces of the stamen. (i, n) Adaxial (i) and abaxial (n) surfaces of the staminodium. (j, o) Ovary wall (j) and stylar (o) surfaces of the carpel. The morphologies of all the organs conform to normal wildtype appearance [32]. Size bars in (a-b) = 2 mm, (c-e) = 1 mm, (f-o) = 50 μm.

In several other taxa, loci in the anthocyanin synthesis pathway have been used as markers for silenced tissue in dual-locus silencing experiments (e.g., [28]). One important criterion for such a marker is that it does not have any effect on other aspects of floral development. *AqANS *is a good candidate in this context since it functions at a late stage in anthocyanin production and, in comparative studies, was frequently found to be down regulated in naturally white flowered species [26]. This may correlate with a lower degree of functional pleiotropy for the locus. We, therefore, carefully examined the morphology of *AqANS*-silenced flowers in order to assess the suitability of the gene as a marker for floral silencing. All floral organ types developed normally in *AqANS*-silenced flowers in terms of overall shape and size. Moreover, no variation from wildtype was observed at the cell level (Figure 8), suggesting that *AqANS *is a useful marker for dual-locus floral silencing experiments.

### Dual TRV-*AqANS*-*AqPI*-VIGS treatment of adult *Aquilegia vulgaris *plants

**Figure 9:**
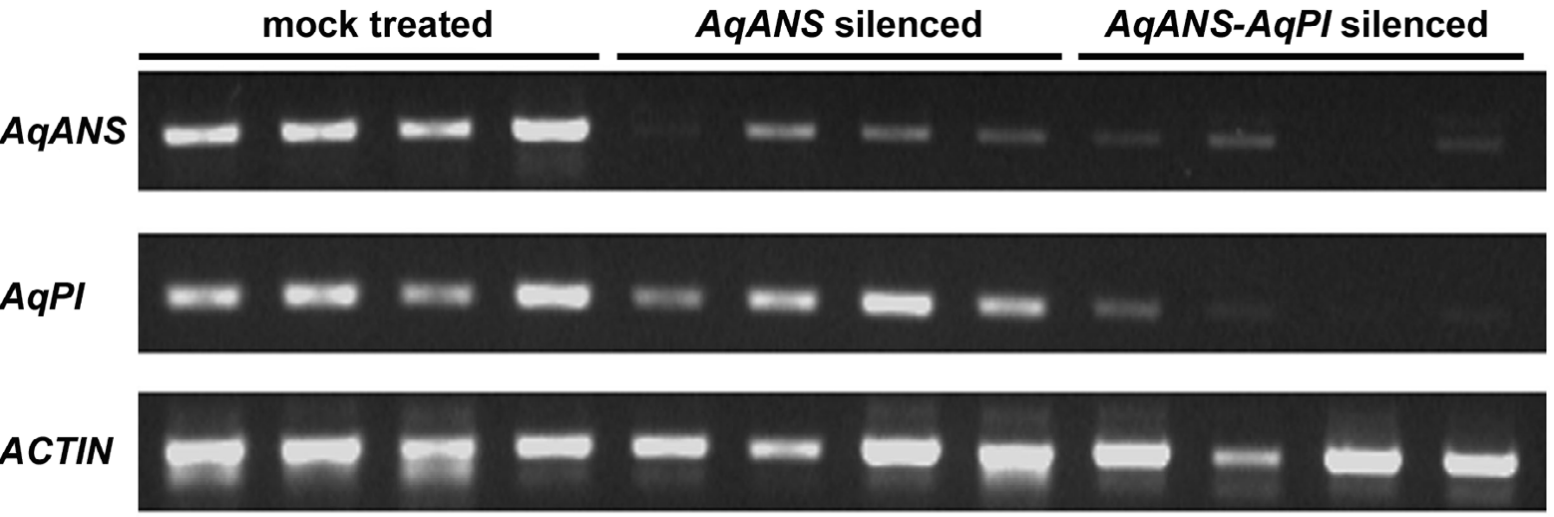
**Dual *AqANS*-*AqPI *silenced flowers**. (a-d) Flowers showing strong silencing of *AqANS *and *AqPI *with correlated reduction in anthocyanin production and homeotic transformation of petals into sepals and stamens/staminodia into carpels.

**Figure 10:**
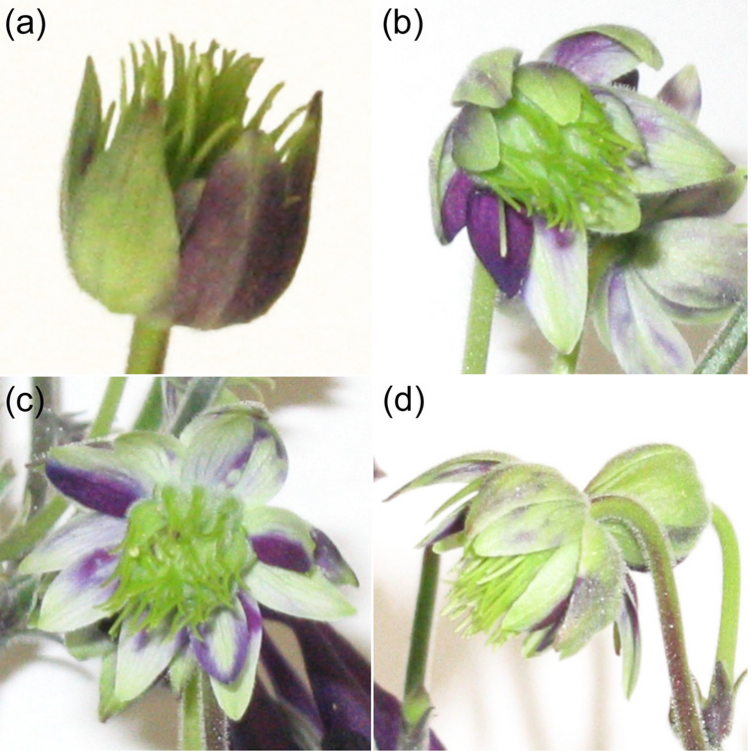
**Endogenous expression levels of *AqANS *and *AqPI***. RT-PCR results for mock TRV1/TRV2 (unmodified) treated, *AqANS *silenced, and *AqANS*/*AqPI *silenced plants. Dual silencing of *AqANS *and *AqPI *is only observed in plants treated with the TRV2-*AqPI*-*AqANS *construct.

For TRV-VIGS to be a truly useful tool for functional analyses in *Aquilegia*, it not only has to be able to knock-down marker genes such as *AqANS *but also reduce specific gene transcript levels with the efficiency and correct timing required to generate discernable mutant developmental phenotypes. It would also be preferable if the method were able to simultaneously silence both a marker gene and an unrelated gene of interest. In order to test these abilities of the TRV-VIGS system in *Aquilegia*, we treated a group of 15 plants with TRV1 and a TRV2-*AqPI-AqANS *construct (Figure 1). This TRV2 construct is designed to silence both *AqANS *and *Aquilegia PISTILLATA (AqPI)*, a MADS box gene normally involved in petal and stamen development [24,25]. Typical loss-of-function phenotypes for *PI *homologs include the homeotic transformation of petals into sepals and stamens into carpels [25,29,30]. In *Aquilegia*, there is an additional organ that may fall under the control of *AqPI *function, the novel staminodium, which is positioned between the stamens and carpels and represents a distinct fifth organ type [31,32]. Dual silencing of *AqANS *and *AqPI *was successful as demonstrated though both RT-PCR and homeotic floral phenotypes (Figures 9 and 10). Flowers showing white sectors also exhibited coordinated transformation of petals to sepals and both stamens and staminodia to carpels (Figure 9). This experiment shows that TRV-VIGS can be used in *Aquilegia *to produce novel gene silencing phenotypes, even in loci that act early in meristem development.

## Conclusion

Establishment of a new model species requires the development of a diverse array of genetic and genomic tools. One critical component in this set of tools is the capacity to perform targeted analyses of gene function. While we are also pursuing the development of stable transformation techniques, the VIGS approach described here has a number of advantages. Among these are the abilities to target specific loci, obtain data with rapid turn around, and potentially knock-down multiple targets that are members of a gene family. In this study, we have demonstrated that TRV-VIGS is an effective tool for silencing gene expression in *A. vulgaris*. To achieve silencing in seedlings, vacuum infiltration with *Agrobacterium *appears to be the most effective approach with 1–2 leaf seedlings showing the strongest response. In addition, it appears that VIGS is suitable for multiple species of *Aquilegia*, perhaps not surprising given its recent radiation [2-4], and can be applied at several different stages of plant development, although at somewhat lower rates of efficiency (data not shown). VIGS in *Aquilegia *can also be used to generate floral developmental phenotypes with low risk of mortality and effective rates of gene silencing. Both a marker gene such as *AqANS *(useful for rapid identification of plants with the strongest levels of silencing) and an unrelated gene of interest can be silenced simultaneously in order to investigate developmental function. The addition of VIGS to our growing set of resources for *Aquilegia *will further advance its rise as an important new model system for the study of evolutionary and ecological questions.

## Methods

### Generation of VIGS TRV2-*AqPDS *and TRV2-*AqANS *constructs

Total RNA was extracted from *Aquilegia vulgaris *leaf tissue using Plant RNA Isolation Reagent (Invitrogen, Carlsbad, CA) and mRNA was purified using Magnetite Oligo (dT) Particles (Novagen (EMD), San Diego, CA). cDNA was generated from 500 ng of mRNA using Superscript II Reverse Transcriptase™ (Invitrogen). The *AqPDS *sequence was isolated through PCR using degenerate primers from conserved regions of the *PDS *gene (PDS-F: 5'-TGGAARGARCAYTCIATGATWTTTGCWATG-3' and PDS-R: 5'-ACRACATGRTACTTIAVDATYTTWGCTTT-3', Figure 1). The primary *AqPDS *fragment was then sequenced to confirm its identity [DQ923721] using BigDye Terminator^® ^(Applied Biosystems, Foster City, CA) and re-amplified using locus-specific primers with appended restriction sites (PDS-F2-XbaI: 5'-GGTCTAGACAGCCGATTTGATTTCCCAGAT-3' and PDS-R3-BamH1: 5'-AAGGATCCGAGAATTGAGTCGGACTTCACC-3', Figure 1). The *AqANS *sequence was obtained from the *Aquilegia *EST database [DR946275 and DR946276], and primers were designed to amplify a 440 bp fragment of the gene (ANS-F-Xba1: 5'-GGTCTAGATTGGGATTGGAAGAAGAAAGGC-3' and ANS-R-BamH1: 5'-AAGGATCCATGTTGAGCAAATGTGCGA-3', Figure 1).

Both gene products were double digested with XbaI and BamH1, as was the TRV2 vector, and ligated separately using T4 DNA ligase (New England Biolabs, Ipswich, MA). The resulting constructs were transformed into heat-shock competent *E.coli *(TOP10 cells, Invitrogen) and plated on selective LB media containing 50 μg/mL kanamycin. Colonies were PCR screened for the presence of the modified constructs using primers 156 F (5'-TTACTCAAGGAAGCACGATGAGC-3') and 156 R (5'-GAACCGTAGTTTAATGTCTTCGGG-3'), which span the multiple cloning site in TRV2. Both constructs were then purified (Fast-Plasmid Mini kit, Eppendorf, Hamburg, Germany) and the identity of the final constructs was verified by sequencing with the 156 F and 156 R primers and BigDye Terminator^®^

### Germination of A. *vulgaris *seedlings and growth of adult plants

When germinating *Aquilegia vulgaris *seed, we found that seeds soaked in distilled water for 24 hours, planted in soil and then stratified at 4°C for at least 3 weeks show the best germination rates. Seeds germinate approximately 10 days after being removed from stratification. More mature seed, stored for 6–8 months at 4°C, had higher germination rates than fresh seed or seed stored for 1–2 months. Treatment of seed with gibberellic acid (GA) at varied concentrations did not improve germination of either fresh or aged seed, however it has been previously reported that seed stored at 20°C may have overall higher germination rates and benefit from GA treatment [33]. Similar rates of germination were observed for seeds planted in all-purpose nursery soil (Fafard 3 B Mix) versus a 1:1 mixture of fine soil and vermiculite. For the TRV2-*AqPDS*-VIGS experiment, *A. vulgaris *seeds (collected from the Harvard University experimental garden) were soaked in distilled water for 24 hours at room temperature and then planted 1/8 inch deep in soil. They were lightly watered, covered, and stratified in the dark for 4 weeks at 4°C. After stratification, they were moved to a growth chamber at 20°C under long days until germination was observed. For the TRV2-*AqANS*-VIGS experiment, seedlings were transplanted and allowed to grow for 12 weeks or more, until they had at least 15 leaves, and then vernalized for 8 weeks either outdoors in winter or in a growth chamber on short days at 4°C. They were then removed to a 20°C chamber and treated 1–2 weeks afterward.

### Preparation of *Agrobacterium*

Electrocompetent cells of *Agrobacterium *strain GV3101 were prepared and transformed as described by Weigel and Glazebrook (2002). *Agrobacterium *and unmodified TRV vectors were kindly provided by S.P. Dinesh-Kumar, Yale University. Transformation was achieved using 1 μL of purified construct DNA per 50 μL competent cells shocked at 2.4 kV using an ECM399 electroporator (BTX Genetronics, San Diego, CA). After 1 hour incubation at 25°C, the cells were plated on selective media (50 μg/mL kanamycin, 50 μg/mL gentamycin, and 25 μg/mL rifampicin) and grown for 3–4 days at room temperature. Colonies were PCR screened using primers 156 F and 156 R for the presence of TRV2-*AqPDS *or TRV2-*AqANS*. Glycerol stocks were made from single positive transformants.

### Vacuum infiltration of TRV1 and TRV2-*AqPDS *constructs into *A. vulgaris*

*Agrobacterium *transformants carrying TRV1 and either TRV2 or TRV2-*AqPDS *were streaked onto selective plates and single colonies were selected for growth in separate liquid cultures (50 μg/mL kanamycin, 50 μg/mL gentamycin, and 25 μg/mL rifampicin). Cultures were sequentially inoculated into a final volume of 550 mL and grown for 72 hours with shaking at room temperature. The cultures were collected by centrifugation at 4000 g for 10–15 minutes and each resuspended to an OD_600 _of approximately 2.0 in an infiltration buffer containing 10 mM MES (2-[N-morpholino]ethanesulfonic acid), 200 μM acetosyringone (3'5'Dimethoxy-4'-hydroxyacetophenone), and 10 mM MgCl_2_. The solutions were then incubated at room temperature for 3–4 hours.

Soil was removed from seedlings with 1–5 true leaves by gently floating them in distilled water. They were then immersed in a 1:1 mixture of the two *Agrobacterium *solutions with added 100 uL/L Silwet L-77 (Lehle Seeds, Round Rock, TX) and infiltrated under vacuum for 2 minutes. For each batch, approximately 15 seedlings were infiltrated per liter of bacterial mixture, with up to 126 seedlings treated per round. The seedlings were drained, transplanted to fresh soil, covered with clear plastic for at least 24 hours, and grown under long days (14 h light, 10 h dark). Over the course of four replicate experiments, a total of 229 *A. vulgaris *seedlings were mock-treated with TRV1 and the empty TRV2 construct, and 406 were treated with TRV1 and the TRV2-*AqPDS *construct.

### Injection of TRV1 and TRV2-*AqANS *into adult *A. vulgaris *plants

Transformed TRV1 and TRV2-*AqANS Agrobacterium *cultures were grown, resuspended, incubated, and combined as described above for the vacuum infiltration procedure, except cultures were only grown for 36 hrs to a volume of 50 mL. 1–2 weeks after adult plants were removed from vernalization, a wound about 1 cm deep was made by applying a clean razor blade to a small area at the base of the basal rosette of each plant, where the leaf bases converge. Approximately 1 mL of *Agrobacterium *mix was injected at the site with a needle-less 1 mL syringe. Depending on the size of the plant and the number of crowns (lateral rosettes), this was done at multiple sites. The procedure was repeated at weekly intervals for 5–6 weeks with new injection sites created with each treatment.

### RT-PCR analyses of viral transcripts and *AqPDS *expression

Leaves were scored for phenotype 5–6 weeks post infiltration. Leaves exhibiting a range of silencing phenotypes were collected and flash frozen at -80°C. Tissue was collected from a total of 96 plants (including untreated plants). Total RNA was extracted from the tissue and purified using the RNeasy Plant Mini kit (Qiagen, Valencia, CA). Each total RNA sample was DNAse treated to remove residual genomic DNA and the concentration was adjusted to 0.3 μg/μL. Three separate cDNA pools were prepared for each RNA sample using 3.3 μL of DNase-treated RNA, Superscript II Reverse Transcriptase (Invitrogen), and reverse primers specific for TRV1 (pYL156 R, [20]), TRV2 (OYL198, [20]), or a poly-T primer [34]. A total of 61 samples were PCR screened for the presence of viral RNAs using primers pYL156F/R (for TRV2, [20]) and OYL195/198 (for TRV1, [20]). To determine relative levels of *AqPDS *expression, we first established the linear range of amplification for *ACTIN *and *AqPDS *in our polyT cDNA samples (25–29 cycles was optimal for *ACTIN *and 28–32 cycles for *AqPDS*) and then used semi-quantitative RT-PCR analysis on cDNAs from 7 to 9 tissue samples per seedling category for a total of 64 samples. *ACTIN *and *AqPDS *were amplified in separate reactions (*ACTIN *for 27 cycles, *AqPDS *for 29 cycles). Primers for the *ACTIN *control were ACT1/2 [20] and for *AqPDS *were PDS-F2 (5'-CAGCCGATTTGATTTCCCAGATGTTCTTCCAGCAC-3') and PDS-R4 (5'-AATCTCTTTCCACTCCTCGGGCAG-3') (Figure 1). Note that due to the G/C rich sequence of the R4 primer region, a longer F2 primer was necessary to achieve comparable T_m _values. PCR reactions were separated by electrophoresis in a 1% agarose gel containing 0.5 mg/L ethidium bromide. DNA band intensities were UV imaged on an Alpha Innotech ChemiImager at levels below saturation and calibrated against a low DNA mass ladder (Invitrogen) using AlphaEase FC imaging software (Alpha Innotech, San Leandro, CA). A subset of reactions was repeated twice for accuracy and the final *AqPDS*/*ACTIN *values were normalized to the lowest ratio.

### RT-PCR analyses of viral transcripts and *AqANS *expression

Floral tissue exhibiting a range of silencing was collected and flash frozen at -80°C 1–2 weeks after the last injection treatment. Tissue was collected from a total of 27 plants, and 52 flowers (including untreated plants). Total RNA was extracted from *Aquilegia combined sepal and petal *tissue using Plant RNA Isolation Reagent (Invitrogen, Carlsbad, CA).). Each total RNA sample was DNAse treated to remove residual genomic DNA. Three separate cDNA pools were prepared for each RNA sample using 0.9 μg of DNase-treated RNA, Superscript II Reverse Transcriptase (Invitrogen), and reverse primers specific for TRV1 (pYL156R), TRV2 (OYL198) or the poly-T primer. A total of 29 samples from 22 separate plants were PCR screened for the presence of viral RNAs as described above.

To determine relative levels of *AqANS *expression, we first established the linear range of amplification for ACTIN and *AqANS *in our polyT cDNA samples, using cDNA diluted 1:200 (30–38 cycles was optimal for *ACTIN *and 27–33 cycles was optimal for *AqANS*). We then performed semi-quantitative RT-PCR analysis on cDNAs from 30 tissue samples (Figure 7). *ACTIN *and *AqANS *were amplified in separate reactions (*ACTIN *for 34 cycles, *AqANS *for 31 cycles). Primers for the *ACTIN *control were ACT1/2, and for *AqANS *were ANS-F2 (5'-AGTTCATTCCCAAGGAGTATGTGC-3') and ANS-R2 (5'-TACTTTTTACCCACTGACGGT-3') (Figure 1). ANS-F2 is outside of the TRV2-*AqANS *insert region (to avoid detecting background plasmid ANS sequences), and the screen region also spans a genomic intron to help avoid detection of any residual genomic *AqANS *sequences (Figure 1). Similar to the *AqPDS *analysis, PCR reactions were separated by electrophoresis in a 1% agarose gel containing 0.5 mg/L ethidium bromide. DNA band intensities were UV imaged on an Alpha Innotech ChemiImager at levels below saturation and calibrated against a low DNA mass ladder. A subset of reactions was repeated twice to test for precision and improve accuracy, and the final *AqANS*/*ACTIN *values were normalized to the lowest ratio.

### Morphological characterization of TRV2-*AqANS *silenced flowers

Floral organs showing strong silencing of *AqANS *were identified based on their bright white color. Light photographs of dissected organs were prepared using a Kontron Elektronik ProgRes 3012 digital camera mounted on a Leica WILD M10 dissecting microscope (Harvard Imaging Center). For scanning electron microscopy (SEM) studies, dissected floral organs were fixed under vacuum in FAA (50% ethanol, 4% formalin, and 5% glacial acetic acid) and then transferred to 70% EtOH for storage. Before imaging, organs were dehydrated through an ethyl alcohol series and critical point-dried with CO_2 _in a Tousimis/Autosamdri-815 dryer. Material was mounted on aluminum stubs with carbon conductive adhesive tabs (Electron Microscopy Sciences, Ft. Washington, PA), sputter-coated with gold palladium in a Denton/Desk II, and studied in a model Quanta 200 SEM (FEI company, Hillsboro, OR, USA) at 5–20 kv.

### TRV2-*AqPI-AqANS *Experiment

The generation of the TRV2-*AqPI*-*AqANS *construct is described in Kramer *et al*. [32]. Culture preparation and injection was performed as described for TRV2-*AqANS*. RT-PCR was performed as described for TRV2-*AqANS *and in Kramer *et al*. [32].

## Competing interests

The author(s) declare that they have no competing interests.

## Authors' contributions

BG participated in the design of the study, conducted all of the experiments described above and drafted the manuscript. EMK conceived of the study, participated in its design and coordination, and helped to draft the manuscript. All authors read and approved the final manuscript.

## References

[B1] Munz PA (1946). Aquilegia: The cultivated and wild columbines.. Gentes Herbarium.

[B2] Hodges SA, Givnish TJ, Sytsma KJ (1997). Rapid radiation due to a key innovation in columbines.. Molecular evolution and adaptive radiation.

[B3] Hodges SA (1997). Floral nectar spurs and diversification.. Int J Plant Sci.

[B4] Hodges SA, Arnold ML (1994). Columbines: a geographically wide-spread species flock.. Proc Nat'l Acad Sci.

[B5] Hodges SA, Whittall JB, Fulton M, Yang JY (2002). Genetics of floral traits influencing reproductive isolation between Aquilegia formosa and Aquilegia pubescens.. Am Nat.

[B6] Institute CUG CUGI Aquilegia Physical Map. http://www.genome.clemson.edu/projects/aquilegia/.

[B7] TIGR TIGR Aquilegia Gene Index. http://compbio.dfci.harvard.edu/tgi/cgi-bin/tgi/gimain.pl?gudb=aquilegia.

[B8] Institute JG JGI Community Sequencing Program FY2007. http://www.jgi.doe.gov/sequencing/why/CSP2007/aquilegia.html.

[B9] Dinesh-Kumar SP, Anandalakshmi R, Marathe R, Schiff M, Liu Y, Grotewold E (2003). Virus-Induced Gene Silencing. Plant Functional Genomics.

[B10] Robertson D (2004). VIGS vectors for gene silencing: Many targets, many tools. Annual Review of Plant Biology.

[B11] Burch-Smith TM, Anderson JC, Martin GB, Dinesh-Kumar SP (2004). Applications and advantages of virus-induced gene silencing for gene function studies in plants. Plant Journal.

[B12] Watson JM, Fusaro AF, Wang MB, Waterhouse PM (2005). RNA silencing platforms in plants.. Febs Journal.

[B13] Hein I, Pacak MB, Hrubikova K, Williamson S, Dinesen M, Soenderby IE, Sundar S, Jarmolowski A, Shirasu K, Lacomme C (2005). Virus-induced gene silencing-based functional characterization of genes associated with powdery mildew resistance in barley. Plant Physiology.

[B14] Scofield SR, Huang L, Brandt AS, Gill BS (2005). Development of a virus-induced gene-silencing system for hexaploid wheat and its use in functional analysis of the Lr21-mediated leaf rust resistance pathway. Plant Physiology.

[B15] Ratcliff F, Martin-Hernandez AM, Baulcombe DC (2001). Tobacco rattle virus as a vector for analysis of gene function by silencing. Plant Journal.

[B16] Hernandez C, Visser PB, Brown DJF, Bol JF (1997). Transmission of tobacco rattle virus isolate PpK20 by its nematode vector requires one of the two non-structural genes in the viral RNA 2. Journal of General Virology.

[B17] VIDE VIDE Database. http://image.fs.uidaho.edu/vide/descr808.htm.

[B18] Liu Y, Nakayama N, Schiff M, Litt A, Irish VF, Dinesh-Kumar SP (2004). Virus induced gene silencing of a DEFICIENS ortholog in Nicotiana benthamiana. Plant Molecular Biology.

[B19] Wang CC, Cai XZ, Wang XM, Zheng Z (2006). Optimisation of tobacco rattle virus-induced gene silencing in Arabidopsis. Functional Plant Biology.

[B20] Hileman LC, Drea S, de Martino G, Litt A, Irish VF (2005). Virus-induced gene silencing is an effective tool for assaying gene function in the basal eudicot species Papaver somniferum (opium poppy). Plant Journal.

[B21] Demmigadams B, Adams WW (1992). Photoprotection and Other Responses of Plants to High Light Stress. Annual Review of Plant Physiology and Plant Molecular Biology.

[B22] Siefermannharms D (1987). The Light-Harvesting and Protective Functions of Carotenoids in Photosynthetic Membranes. Physiologia Plantarum.

[B23] Holton TA, Cornish EC (1995). Genetics and biochemistry of anthocyanin biosynthesis.. Plant Cell.

[B24] Goto K, Meyerowitz EM (1994). Function and regulation of the Arabidopsis floral homeotic gene PISTILLATA. Genes and Development.

[B25] Bowman JL, Smyth DR, Meyerowitz EM (1989). Genes directing flower development in Arabidopsis.. The Plant Cell.

[B26] Whittall JB, Voelckel C, Kliebenstein DJ, Hodges SA (2006). Convergence, constraint and the role of gene expression during adaptive radiation: floral anthocyanins in Aquilegia. Mol Ecol.

[B27] Clough SJ, Bent AF (1998). Floral dip: a simplified method for Agrobacterium-mediated transformation of Arabidopsis thaliana.. Plant J.

[B28] Chen JC, Jiang CZ, Gookin TE, Hunter DA, Clark DG, Reid MS (2004). Chalcone synthase as a reporter in virus-induced gene silencing studies of flower senescence. Plant Molecular Biology.

[B29] Vandenbussche M, Zethof J, Royaert S, Weterings K, Gerats T (2004). The duplicated B-class heterodimer model: Whorl-specific effects and complex genetic interactions in Petunia hybrida flower development.. Plant Cell.

[B30] Trobner W, Ramirez L, Motte P, Hue I, Huijser P, Lonnig WE, Saedler H, Sommer H, Schwarz-Sommer Z (1992). Globosa - a homeotic gene which interacts with deficiens in the control of antirrhinum floral organogenesis. EMBO J.

[B31] Tucker SC, Hodges SA (2005). Floral ontogeny of Aquilegia, Semiaquilegia, and Isopyrum (Ranunculaceae).. Int J Plant Sci.

[B32] Kramer EM, Holappa L, Gould B, Jaramillo MA, Setnikov D, Santiago P (2007). Elaboration of B gene function to include the identity of novel floral organs in the lower eudicot Aquilegia (Ranunculaceae).. Plant Cell.

[B33] Deno NC (1993). Seed Germination: Theory and Practice, 2nd edition..

[B34] Kramer EM, Dorit RL, Irish VF (1998). Molecular evolution of genes controlling petal and stamen development: Duplication and divergence within the APETALA3 and PISTILLATA MADS-box gene lineages.. Genetics.

